# Icariin Treatment Rescues Diabetes Induced Bone Loss via Scavenging ROS and Activating Primary Cilia/Gli2/Osteocalcin Signaling Pathway

**DOI:** 10.3390/cells11244091

**Published:** 2022-12-16

**Authors:** Jie Liu, Qingfeng Cheng, Xiangmei Wu, Huifang Zhu, Xiaoyan Deng, Maorong Wang, Shengyong Yang, Jie Xu, Qian Chen, Mengxue Li, Xianjun Liu, Changdong Wang

**Affiliations:** 1 Department of Biochemistry and Molecular Biology, Molecular Medicine and Cancer Research Center, College of Basic Medicine, Chongqing Medical University, Chongqing 400016, China; 2 Department of Endocrinology, The First Affiliated Hospital of Chongqing Medical University, Chongqing 401331, China; 3 Department of Physiology, Molecular Medicine and Cancer Research Center, College of Basic Medicine, Chongqing Medical University, Chongqing 400016, China; 4 Department of Endocrinology, Affiliated Hospital of Hubei University for Nationalities, Enshi 445000, China

**Keywords:** diabetes, bone loss, primary cilia, Icariin, ROS

## Abstract

Diabetes-associated bone complications lead to fragile bone mechanical strength and osteoporosis, aggravating the disease burden of patients. Advanced evidence shows that chronic hyperglycemia and metabolic intermediates, such as inflammatory factor, reactive oxygen species (ROS), and advanced glycation end products (AGEs), are regarded as dominant hazardous factors of bone complications, whereas the pathophysiological mechanisms are complex and controversial. By establishing a diabetic Sprague-Dawley (SD) rat model and diabetic bone loss cell model in vitro, we confirmed that diabetes impaired primary cilia and led to bone loss, while adding Icariin (ICA) could relieve the inhibitions. Mechanistically, ICA could scavenge ROS to maintain the mitochondrial and primary cilia homeostasis of osteoblasts. Intact primary cilia acted as anchoring and modifying sites of Gli2, thereby activating the primary cilia/Gli2/osteocalcin signaling pathway to promote osteoblast differentiation. All results suggest that ICA has potential as a therapeutic drug targeting bone loss induced by diabetes.

## 1. Introduction

Diabetes mellitus (DM) is a global disease which severely threatens human health after cardiovascular diseases and tumors. Statistically, IDF studies in the 20–79-year old age group show that approximately 536.6 million people worldwide had diabetes (prevalence 10.5%) in 2021, and this is projected to reach 783.2 million people in 2045 (prevalence 12.2%) [1]. Particularly in middle-income countries, the incidence of diabetes is expected to grow the fastest, reaching 21.1%, showing a rapid rise in incidence to epidemic levels and a trend toward younger patients [2,3]. Compared with healthy people of the same age, diabetic patients have 2–10 times higher risk of death due to cardiovascular diseases, such as coronary heart disease and stroke, and the risk of suffering from retinopathy and bone microvascular complications is increased as well [4].

As the population continues to age and underlying diseases, such as endocrine, chronic inflammatory, and genetic disorders occur, more than 18.3 million fractures occur each year in the United States alone [5], with the high incidence of diabetes being an important predisposing factor for increased bone injury disease [6]. The bone mineral density (BMD) of type 1 diabetes mellitus (T1DM) patients is significantly decreased, regardless of the gender [7]. It has been hypothesized that the absence of anabolic effects of insulin and other factors lead to low peak bone mass, because T1DM frequently manifests before peak bone mass [6]. However, the paradox is increased bone fragility in type 2 diabetes mellitus (T2DM) patients despite normal or even increased BMD [8], perhaps due to low efficiency of bone turnover and inferior microarchitecture (particularly increased cortical porosity and low cortical volumes) [9].

In both patients with type T1DM and T2DM, the mechanical strength of bone is declining, and the risk of fracture is on the rise [10]. The bone remodeling is crucial for continuous bone turnover and renewal depending on synergistic effects between bone-resorbing osteoclasts and bone-forming osteoblasts [6]. However, the diabetics appear decreased capabilities of bone turnover which inevitably results in increased fracture risk and delayed healing [11]. The pathophysiological mechanism that induces diabetic bone fragility is fairly complex, including dysfunction of signaling pathway (such as insulin and Wnt signaling) and the accumulation of harmful factors, such as inflammatory factors, microvascular impairment, and AGEs, which destroy the collagen structure, increase fat content in bone marrow, and change the function of osteocytes [6,12]. Furthermore, disordered ratio of OPG/RANK/RANKL breaks bone homeostasis [13]. Although more than 20 years of intensive research has seen numerous hypotheses, the specific mechanism of diabetes-related bone complications remains unclear.

Reactive oxygen species (ROS), the main productions of oxidative stress, remain ambivalent because they are both essential and detrimental to life; the tipping point depends on concentration [14]. A large body of evidence has confirmed that long-term hyperglycemia could provoke mitochondria, eliciting excessive ROS. These organelles are subject to the deleterious effects of ROS themselves and eventually result in dysfunctional organs, especially the cardiovascular system, brain, and kidney [15]. Seminal discoveries have deconstructed the bone microenvironment into a unique niche composed of multiple cellular and extracellular components and shown the devastating influence of the diabetic microenvironment on bone metabolism [16]. Osteoblasts play a central role in bone formation, while ROS also reduce osteoblast activity and functional response [17]. Ma et al. found that ROS accumulation in type 2 diabetes led to mitochondrial dysfunction and ferroptosis in osteoblasts [18]. Recently, substantial experiments on pharmacological treatment of diabetic bone damage have focused on ROS scavenging as a therapeutic route [16,19], suggesting the possibility of targeting ROS to inhibit downstream deleterious metabolic pathways for therapy of diabetic bone complications.

Osteoporosis can be prevented and treated, as therapeutic agents have evolved from initial estrogen and calcitonin to current bisphosphonates, RANKL antibody, parathyroid hormone analogue, sclerostin antibody, and Cathepsin K inhibitor [20]. Although remarkable advances have been achieved along with the development of therapeutic drugs, we cannot neglect the side effects and ambiguous long-term effects of these drugs. This secondary osteoporosis induced by diabetes may not respond adequately to conventional anti-osteoporotic drugs if the underlying condition is not identified and treated [21]. Therefore, in order to alleviate patients’ worries about drug safety and ensure getting seasonable treatment, we should find novel therapeutic drugs from other fields. *Epimedium*, a traditional Chinese herb, has been used for treating fractures and osteoporosis for long time [22]. Icariin (ICA), the purified extract of *Epimedium*, could relieve the inhibition of PDE5 on aromatase P450 and promote the catalytic synthesis of estrogen from C19 androgen [23]. Animal experiments have shown that ICA functioned as an estrogen analog to protect ovariectomized rats by increasing osteogenesis and angiogenesis [24]. Here, the underlying mechanism is that ICA can activate ERα/Akt by inducing IGF-1 production to promote bone formation [25]. Cell experiments have shown that ICA could increase the osteoblast differentiation and mineralization of bone marrow mesenchymal stem cells (BMSCs) as well as inhibit the formation of osteoclasts and bone resorption [26,27]. In addition, ICA has attracted much attention due to ameliorating lipid metabolism disorders, inflammation, insulin resistance, and mitochondrial dysfunction [28,29]. Ding et al. proposed that ICA could promote Sesn2-induced mitophagy to inhibit NLRP3 inflammasome activation in diabetic nephropathy SD rats [30]. The above results indicate that ICA with various biological activities may play an intriguing role in the treatment of diabetic osteoporosis.

Shi et al. confirmed that ICA could enhance cAMP/PKA/CREB signals in the primary cilia to promote the maturation and mineralization of osteoblasts [31]. In evolution, primary cilia are strictly conserved organelles that act as information transmission hubs and are observed in osteocytes, osteoblasts, and chondrocytes [32]. However, abnormal ciliary morphology has been observed in bone defects, such as short rid-polydactyly syndromes (SRPS) and Jeune asphyxiating thoracic dystrophy (JATD). Kluth et al. found that the number of primary cilia of pancreatic islet cells in diabetic rat models was significantly reduced [33], which may be related to the high levels of inflammatory factors and ROS [34]. A prolonged period of high levels of TGF-β in diabetic patients could induce the expression of HDAC6, which deacetylates α-tubulin protein, leading to cilia shortening and even degradation, thereby preventing the maturation of osteoblasts [35]. Current findings indicate that the abnormalities of primary cilia caused by diabetes involves multiple tissues and organs. However, little research has been performed on bone damage caused by the destruction of primary cilia by diabetes, and whether this damage can be treated by protecting primary cilia is largely unknown.

Based on current research results, we imply primary cilia could respond to hyperglycemia by disassembly, which is an important factor leading to diabetes-related bone loss. Our findings confirmed that the reduced number of primary cilia in osteoblasts led to the inhibition of differentiation, which was linked to the excess ROS caused by diabetes. ICA could act as a ROS inhibitor to maintain the mitochondrial and primary cilia homeostasis of osteoblasts and activate the primary cilia/Gli2/osteocalcin pathway, thereby rescuing bone loss. These results provide insight into the underlying mechanism of diabetic bone complications and suggest a potential therapeutic target for future treatment.

## 2. Materials and Methods

This study was conducted in accordance with the Declaration of Helsinki and was approved by the Ethics Committee of Affiliated Hospital of Hubei University for Nationalities (Grant No. P2022006). The patients and their families are informed of this study and have signed informed consent. All studies and operations were performed on rats according to the procedures approved by Institutional Animal Care and Use Committee of Chongqing Medical University.

### 2.1. Reagents and Antibodies

Icariin (Chengdu Plant Pharmaceutical Factory, purity > 98%, Chengdu, China), α-MEM medium, Fetal Bovine Serum (Gibco, New York, NY, USA), Alizarin Red (Solarbio, Beijing, China), Mito-Tracker Green(C1048, Beyotime, Shanghai, China), alkaline phosphatase kit (NPP substrate-AMP buffer method), Glucose measuring kit (Glucose oxidase method) (BIOSINO BIO-TECHNOLOGY, Beijing, China), OCN ELISA kit (MEB1616, Mengbio, Chongqing, China), DCFH-DA (2′,7′-Dichlorodihydrofluorescein diacetate, E004, Nanjing Jiancheng Bioengineering Institute, Nanjing, China), PGC-1α (1:1000, D162041), TFAM (1:1000, D154208), Streptozotocin, NRF1(1:1000, D261978) (Sangon Biotech, Shanghai, China), Cyclopamine (C4116), β-glycerophosphate, dexamethasone, ascorbic acid, acetylated α-tubulin antibody (1:1000, T6793), γ-tubulin antibody (1:1000, T3320), cetylpyridinium chloride (Sigma, Saint Louis, MO, USA), Penicillin-Streptomycin Solution (Hyclone, Logan, UT, USA), OPN (1:1000, 225952-1-AP, Proteintech, Rosemont, IL, USA), ALP (1:1000, A5111), SHH (1:1000, A5115) (Selleckchem, Houston, TX, USA), collagen Ⅰ (1:1000, ab255809), OCN (1:1000, ab133612), Runx2 (1:1000, ab54868) (abcam, Cambridge, UK), Ptch1 (1:1000, C53A3), Sufu (1:1000, C54G2), HRP-linked goat anti-rabbit IgG antibody (1:5000, #7074) and HRP-linked goat anti-mouse IgG antibody (1:5000, #7076) (CST, Danvers, MA, USA), Gli2 (1:1000, YX3016), GAPDH (1:1000, YT5052, ImmunoWay, Plano, TX, USA), Alexa Fluor 568 conjugated anti-rabbit antibody, Alexa Fluor 647 conjugated anti-mouse antibody (Invitrogen, Carlsbad, CA, USA).

### 2.2. T1DM SD Rats Model Construction

SD rats aged 8 weeks were purchased from experimental Animal Center of Chongqing Medical University. The streptozotocin had toxic effects on pancreatic islet β-cells specially, causing almost all pancreatic β-cell necrosis [36]. Then STZ was dissolved into a sodium citrate buffer (50 mM, pH = 4.5) at a final concentration of 100 mg/mL. A T1DM SD rat model was established by intraperitoneally injecting at a single large dose STZ (1 mL/kg, diabetes group) or sodium citrate buffer (1 mL/kg, vehicle control group) [37]. After a week, rats with random blood glucose concentration higher than 250 mg/dL (13.89 mmol/L) could be defined as T1DM. Referring to the methods of Tao et al., we maintained the diet for 90 days to study the effects of diabetes on bone [17].

### 2.3. Diabetic Cell Model Constructionin Vitro

#### 2.3.1. Primary Osteoblasts Isolation, Culture and Osteoblast Differentiation Induction

Referring to the previous report [38], in a sterile environment, bone marrow mesenchymal stem cells (BMSC) and osteoblasts derived from newborn rat calvaria (primary OB) were isolated from 3–4-day old euthanized rats and were plated inα-MEM medium supplemented with 10% fetal bovine serum and 1% Penicillin-Streptomycin solution at 37 °C in a 5% CO_2_ humidified incubator. Cells were selected at passage 3 to seed into 6-well plates at density of 1 × 10^5^ cells per well. When confluence reached 90%, cells were induced by osteoblast differentiation α-MEM medium (containing 10% FBS, 1%Penicillin-Streptomycin solution, 10 mM β-glycerophosphate, 1 × 10^−8^ M dexamethasone and 50 μg/mL ascorbic acid).

#### 2.3.2. Cell Model Construction

We referred to the method of Hernández et al. [39] and utilized three different osteoblast cell lines, namely BMSC, primary OB, and MC3T3-E1 to construct the cell model of diabetes in vitro, by means of adding D −( + )− glucose to medium to simulate a high glucose environment. Osteoblasts were induced by osteoblast differentiation α-MEM medium supplemented with D −( + )− glucose to final glucose concentration of 25 mM (HG group) or normal osteoblast differentiationα-MEM medium (NC group), respectively. Moreover, to study the effects of ICA on osteoblast differentiation, ICA was supplied at final concentrations of 40 μmol/L (optimal concentration, determined by preliminary experiment) into 25 mM group (25 mM + ICA). To study the effects of ICA on Hedgehog signaling, Cyclopamine (CYC) was supplemented at final concentrations of 5 μmol/L into 25 mM + ICA group (25 mM + ICA + CYC), and to study the effects of ICA on ROS, Mito-Tempo was supplied at final concentrations of 5 μmol/L into 25 mM group (25 mM + Mito). After 3 days of induction, total protein was extracted, immunofluorescence was performed, and ROS was determined. After 21 days of induction, mineralized nodules were detected.

#### 2.3.3. Alizarin RED Staining Mineralized Nodules

Alizarin red could specifically stain Ca^2+^-rich substances, so we induced three osteoblast cell lines by osteoblast differentiation medium with different glucose concentrations for 21 days, removed medium, washed with PBS for 3 times, fixed with 4% cold paraformaldehyde for 10 min, then discarded, washed with PBS for 3 times, stained with 0.2% alizarin red solution (pH 8.3) at room temperature, washed with ddH_2_O for 2 times, and scanned stained cells. Then, cells were destained with 10% (*w*/*v*) cetylpyridinium chloride for 30 min. The 100 μL destaining solution was transferred to a 96-well plate and optical density (*a* value) was measured at 562 nm.

#### 2.3.4. Intracellular ROS Determination

Osteoblasts were induced for 3 days and collected cell lysates, DCFH-DA (2′,7′-Dichlorodihydrofluorescein diacetate) probe, and H_2_O_2_ were diluted at 1:1000. The measurement system consisted of 4 μL protein, 46 μL ddH_2_O, 100 μL diluted DCFH-DA and 100 μL diluted H_2_O_2_ solution. Avoiding light and incubating for 30 min at 37 °C, fluorescence intensity was detected according to FITC procedures.

#### 2.3.5. ALP Activity Analysis

Referring to the methods of Xu et al. [38], ALP activity was determined using an ELISA kit according to the manufacturer’s instructions. Briefly, total cell protein was extracted after 3 days of osteoblast differentiation induction. The measurement system consisted of 200 μL working solution and 4 μL cell protein solution. After mixing, cells were incubated at 37 °C and A value was measured at 410 nm for 5 consecutive times at 1-min intervals. Subsequently, we calculated the rate of change of the A value per minute ($\frac{\Delta A}{min}$). ALP activity was determined according to the formula $\left(2.75\times 10^{6}\times \frac{\Delta A}{min}\times 0.06\;\right)U/L\;$.

### 2.4. Immunofluorescence

#### 2.4.1. Primary Cilia Immunofluorescence

MC3T3-E1 cells were plated on sterile glass coverslips at density of 4 × 10^4^ cells/well in 24-well plate. When confluence reached 70%, cells were induced for 3 days. Medium was discarded and cells were washed 3 times with PBS buffer (pH = 7.4), then fixed with 4% paraformaldehyde for 10 min. Fixed cells were permeabilized with 0.05% Triton X-100 and then washed 3 times with PBS. Cells were then incubated with 5% BSA to block non-specific antigen sites for 60 min, and then incubated with antibodies of anti-Acetylated-α-tubulin and anti-γ-tubulin overnight at 4 ℃ followed by Alexa Fluor568-conjugated anti-rabbit and Alexa Fluor647-conjugated anti-mouse antibodies were used as secondary antibody. A Leica DM4000 microscope was used to observe and photograph the cells. Ten fields per coverslip (three coverslips per group) were randomly selected.

#### 2.4.2. Mitochondrial Mito-Tracker Green Immunofluorescence

Mito-Tracker Green and DAPI were diluted by serum-free medium at 1:5000 and 1:1000, respectively, and incubated at 37 °C in advance. BMSC, primary OB, and MC3T3-E1 cells were plated on sterile glass coverslips, respectively, at density of 4 × 10^4^ cells/well in 24-well plates. When confluence reached 70%, cells were treated for 3 days. Medium was discarded and cells were washed 3 times with serum-free medium, supplement with 500 μL preheating Mito-Tracker Green solution, and then incubated at 37 °C for 30 min. Medium was again discarded, cells were washed 3 times, and 200 μL DAPI solution was added before incubation at 37 °C for 40 min. A Leica DM4000 microscope was used to observe and photograph the cells. Ten fields per coverslip (three coverslips per group) were randomly selected.

### 2.5. Plasmids and Transfection

Full-length mouse OCN (BGLAP2, Gene ID: 12097) cDNA was cloned and ligated into pcDNA3.1 (+) vector after digested by BamHⅠ/XhoⅠ restriction enzyme. The plasmid of pcDNA3.1-Gli2 was generously provided by Professor Zhang Ying, Chongqing Medical University. To confirm expression of the introduced coding sequences, HEK-293T cells were transfected with the expression plasmid using the transfection reagent Lipofectamine 2000 (Invitrogen) according to the protocol. Briefly, 2 × 10^5^ HEK-293T cells were placed into each well of a 6-well plate. When confluence reaches 80%, cells were transfected with corresponding plasmids (pcDNA3.1-OCN, pcDNA3.1-Gli2 and empty vector). Expression was confirmed by detecting protein extracts of these cells with ELISA kit and western blot.

### 2.6. Immunohistochemistry

Femur and tibia tissues dissected from SD rats were fixed using 4% cold paraformaldehyde for 48 h and decalcified in 10% EDTA (pH 7.4) for 60 days at room temperature. Subsequently, paraffin-embedded tissues were sawed along the largest section of the sagittal plane (5 μm thick), and sections were collected on charged glass slides for H&E staining and immunofluorescence. The pancreases of each group were surgically removed and fixed in 4% paraformaldehyde for 48 h, then made into paraffin sections for H&E staining and immunofluorescence.

### 2.7. Micro-Computed Tomography (Micro-CT)

Micro-CT was performed to evaluate bone mechanical properties and microarchitecture. Hence, 4% paraformaldehyde fixed left femur was scanned using a Micro-CT scanner (vivaCT40, SCANCO Medical AG, Zurich, Switzerland). Trabecular bone thickness parameters are calculated by distance transformation (Direct-No Model) and surface triangulation (TRI-Plate Model) methods. BV, TV, BV/TV, Tb.Th, Tb.N, and Tb.Sp were calculated from the region of interest (ROI). X-ray tube: micro focus tube supporting conical X-ray beam, focal diameter of 5 μm. The data were analysed by instrument software (vivaCT40, SCANCO Medical, Zurich, Switzerland).

### 2.8. Western Blot

The protein was denatured in 6 × loading buffer and separated in 10% SDS-PAGE gel. The protein was transferred to the PVDF membrane in a buffer solution of 0.192 mol/L glycine, 0.25 mol/L Tris, and 20% methanol. After blocking with 5% defatted milk-TBST for 1 h, the membrane was incubated with primary antibody overnight at 4 °C, and then incubated with HRP-conjugated goat anti-rabbit/mouse IgG antibody for 1 h. Target protein signal was detected with Western Bright ECL chemiluminescence reagent, and visualized was performed using BIO RAD ChemiDoc™ Touch Imaging System (USA). GAPDH was used as a loading control, and the gray values of protein bands were measured using ImageJ software (Win 64-bit, National Institutes of Health, Bethesda, MD, USA).

### 2.9. Transmission Electron Microscopy (TEM)

Blocked SD rat pancreas approximately 1 mm^3^ in size were double-fixed with 2.5% glutaraldehyde and 1% osmium acid at 4 ℃ overnight. Dehydration was done in grades of ethanol (50%, 70%, 90%) and 90% acetone, and specimens were embedded in Epon812 epoxy resin. Ultrathin sections (50 nm) were mounted on copper grids and contrasted with uranyl acetate and lead acetate. A transmission electron microscope (JEM-1400PLUS, JEOL, Tokyo, Japan) was used to observe and capture images.

### 2.10. Statistical Analysis

All data are presented as mean ± SD. (*n* ≥ 3). Student’s t-test are used to comparison between two groups and one-way ANOVA was used for comparison between the three groups. *p* < 0.05 was used as a threshold for statistical significance. GraphPad Prism was used to visualize data.

## 3. Results

### 3.1. The Serum ALP and P Ion Are Important Indicators Reflecting Diabetic Bone Metabolism Disorders

**Figure 1:**
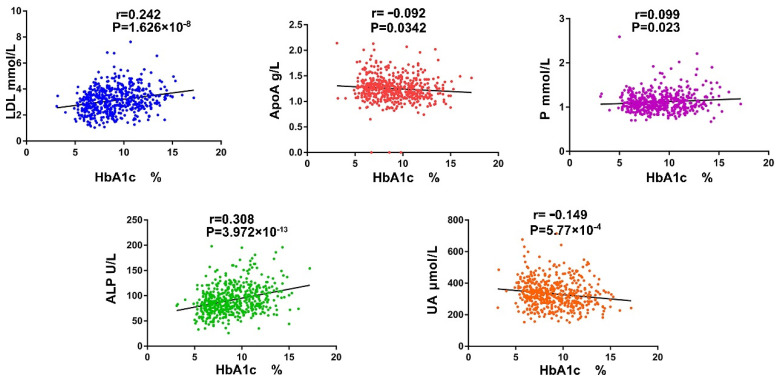
The case data from of T2DM patients. Pearson correlation analysis between HbA1c and serum low density lipoprotein (LDL), ApoA, P ion, ALP and uric acid (UA).

Compared with FBG (fasting blood-glucose), hemoglobin A1c (HbA1c) has an excellent role in the diagnosis and screening of bone injury in diabetic patients [7,40]. We collected the date of 543 T2DM patients (male: 374, female: 169, age: 23–80 years old) to evaluate the correlation between several bone metabolic-related markers and HbA1c. Among them, alkaline phosphatase (ALP), a marker of osteoblast differentiation, was the most positively correlated with HbA1c (r = 0.308) (Figure 1). Subsequently, multiple linear regression analysis of the factors contributing to the deterioration of HbA1c confirmed that ApolipoproteinA (ApoA) and phosphonium ion (P) were the main risk factors. The model accounted for approximately 20% of the variance of HbA1c (R^2^ = 0.203, Adjusted R^2^ = 0.195) (Table 1). ALP and P are important indicators which reflect bone metabolism, and these results indicate that well-controlled HbA1c is important for delaying bone loss.

### 3.2. Diabetes Results in Severe Impairment of Bone Mechanical Properties in SD Rats

**Figure 2:**
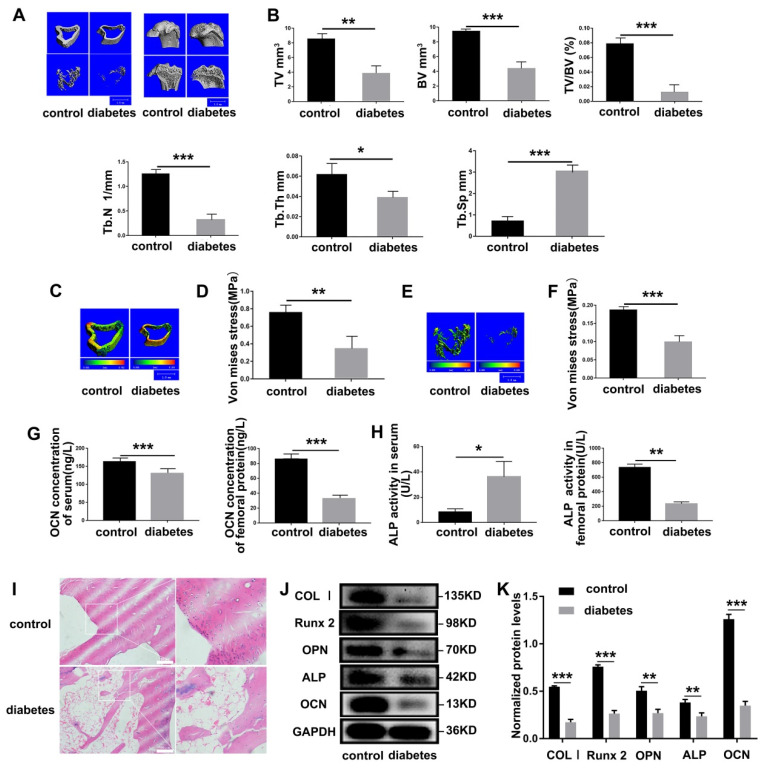
Diabetes cause severe bone defects. (**A**) Micro-CT showed the results of transverse section and longitudinal section of the distant femur. (**B**) Quantitative analysis Micro-CT-derived bone microstructural parameters. (**C**) A comparison of the stress levels of the cortical bone. (**D**) Quantitative analysis bone stress levels of (**C**). (**E**) A comparison of the stress levels of the cancellous bone. (**F**) Quantitative analysis bone stress levels of (**E**). (**G**,**H**) ELISA kit assayed serum and femur OCN concentration and ALP activity. (**I**) H&E staining performed on the distal tibia tissues. (**J**) Western blot analysis of the protein expressions of COL Ⅰ, Runx2, OPN, ALP and OCN in femur. (**K**) Quantitative analysis the expression levels of (**J**). * *p* < 0.05, ** *p* < 0.01 and *** *p* < 0.001, compared with vehicle control SD rats.

Superior bone mechanical properties are the basis for bones to support movement and protect important organs [41]. We utilized Micro-CT to explore the effects of diabetes on the bone microarchitecture. Compared with the control group, diabetic SD rats total tissue volume (TV), bone volume (BV), bone volume fraction (BV/TV), bone trabecular number (Tb.N), and trabecular thickness (Tb.Th) were significantly reduced, while trabecular separation (Tb.Sp) was increased significantly (Figure 2A,B). Meanwhile, the biomechanical detection of femur found that the mechanical strength of cortical bone (Figure 2C,D) and cancellous bone (Figure 2E,F) was significantly weakened, confirming that diabetes caused damage to the mechanical properties of bone. ALP and osteocalcin (OCN) are markers of osteoblast differentiation [42]. Hence, we used ELISA kits to detect these two marker proteins and found that serum OCN, femoral OCN, and ALP were significantly reduced in diabetic SD rats (Figure 2G,H). Interestingly, serum ALP was significantly increased (Figure 2H), which was consistent with previous clinical data. H&E staining performed on the distal tibia showed that diabetes caused osteopenia (Figure 2I). To ensure the robustness of the above results, the osteoblast differentiation markers in femur protein were detected and found reducing protein levels of type I collagen (COL I), runt-related transcription factor 2 (Runx2), osteopontin (OPN), ALP, OCN (Figure 2J,K).

### 3.3. ICA Rescue Osteoblast Differentiation Inhibited by High Glucose

**Figure 3:**
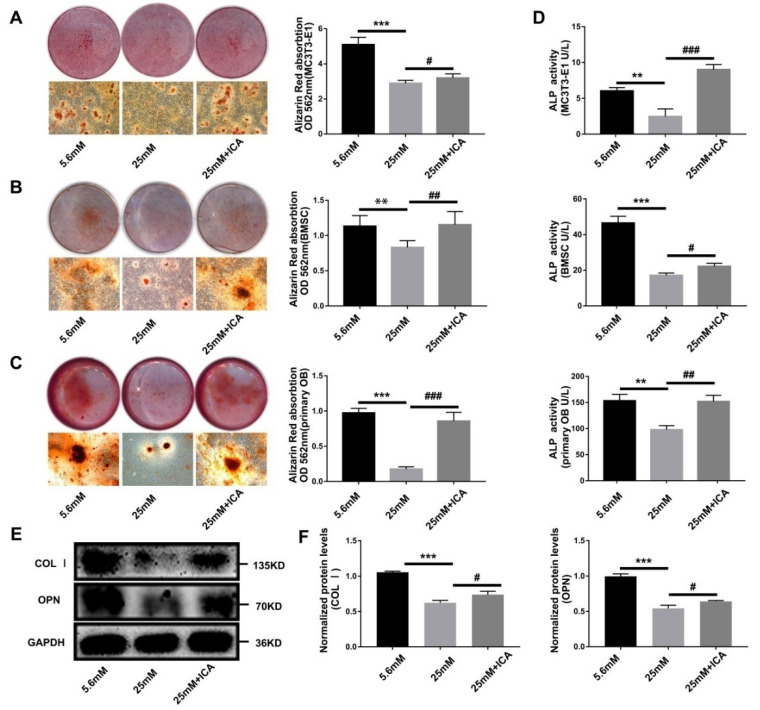
ICA plays a therapeutic role in diabetic bone loss. Three different osteoblast cell lines were induced in for 21 days, and the extracellular matrix mineralized nodules were stained with Alizarin Red. The results of Alizarin red staining and quantitative analysis of MC3T3-E1 (**A**), BMSC (**B**) and primary OB cell (**C**). Three different osteoblast cell lines were induced in different conditions for 3 days, extracted total cell protein to analyze by ELISA kit and western blot. (**D**) ALP activity assay of total cell protein of osteoblasts. (**E**) Western blot analyzed the expression of COL Ⅰ and OPN. (**F**) Quantitative analysis the level of protein expression in (**E**). ** *p* < 0.01 and *** *p* < 0.001, compared with 5.6 mM group. ^#^ *p* < 0.05, ^##^ *p* < 0.01 and ^###^ *p* < 0.001, compared with 25 mM group.

To interrogate the effect of diabetes on osteoblast differentiation and the therapeutic prospect of ICA for diabetic bone loss, we establish diabetic cell model in vitro. Alizarin red staining results showed that the formation of mineralized nodules in high glucose environment (25 mM group) was significantly reduced, while adding 40 μmol/L ICA significantly recovered mineralization (25 mM + ICA group, Figure 3A–C). Meanwhile, we used ELISA kit to assay the ALP activity of extracted total cell protein, and further proved the activity of ALP inhibited by high glucose was increased by the ICA addition (Figure 3D). Then, protein levels analysis of COL Ⅰ and OPN further confirmed the above results (Figure 3E,F). Based on successfully constructed cell models, we found that ICA could relieve the inhibition of osteoblast differentiation and mineralization caused by high glucose.

### 3.4. ICA Can Protect Osteoblast Primary Cilia from High Glucose Damage

**Figure 4:**
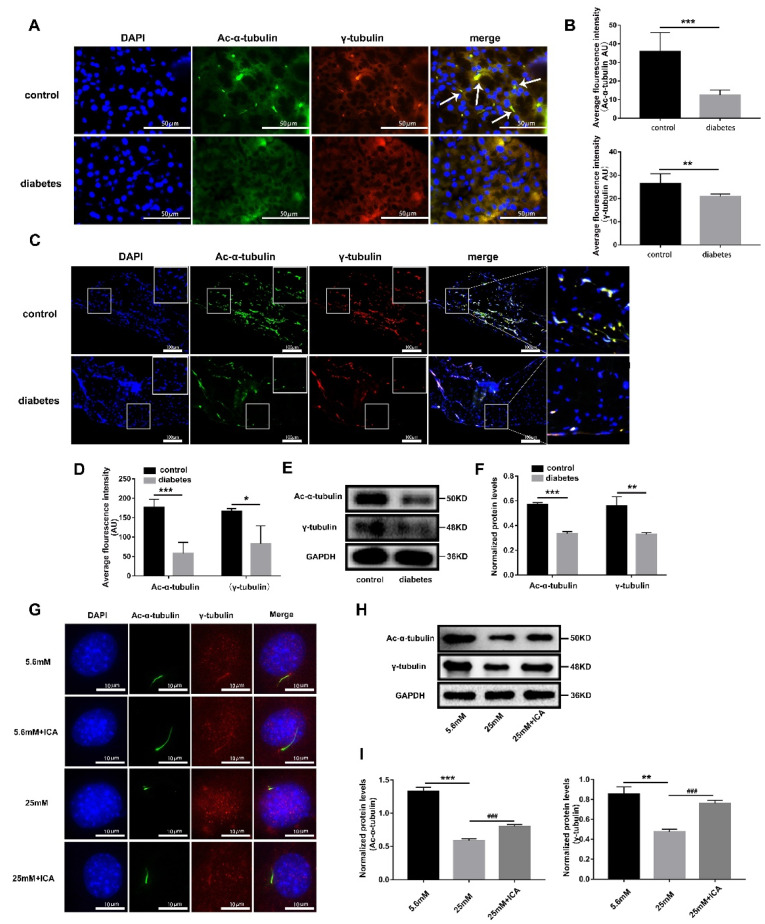
Diabetes impairs primary cilia in bone tissue, and ICA could protect the primary cilia from high glucose damage. (**A**) Immunofluorescence of pancreas, scale bar: 50 μm. (**B**) Quantitative analysis average fluorescence intensity of Ac-α-tubulin and γ-tubulin in (**A**). (**C**) Immunofluorescence of femur tissue, scale bar: 100 μm. (**D**) Quantitative analysis average fluorescence intensity of Ac-α-tubulin and γ-tubulin in (**C**). (**E**) Western blot detected the expression of Ac-α-tubulin and γ-tubulin in the femoral protein. (**F**) Quantitative analysis the level of protein expression in (**E**). ICA was added to the diabetic bone loss cell model and induced for 3 days, (**G**) immunofluorescence observed primary cilia. (**H**) Extracted cell total protein, Western blot detects protein expression of Ac-α-tubulin and γ-tubulin. (**I**) Quantitative analysis the level of protein expression in (**H**). * *p* < 0.05, ** *p* < 0.01 and *** *p* < 0.001, compared with 5.6 mM group. ^###^ *p* < 0.001, compared with 25 mM group.

Primary cilium is a signal transmission hub, which can play a vital role in bone development. There are numerous specific receptors and ion channel in ciliary membrane enabling to sense osmotic pressure, hydrostatic pressure, Ca^2+^ flux and other physical stimuli, and regulate paracrine through signaling pathways, such as Hedgehog, Wnt, and cAMP/PKA/CREB [43], thereby regulating osteoblast proliferation, differentiation, and matrix deposition [44]. Consistent with Kluth’s report [33], we found almost all the primary cilia were missed in pancreas of diabetic SD rats (Figure 4A) compared with the vehicle control group, and the average fluorescence intensity of ciliary structural proteins acetylated α-tubulin (Ac-α-tubulin) and γ-tubulin were significantly reduced (Figure 4B). According to available evidence, it is assumed that diabetes would impair primary cilia of bone tissue. Our results supported this hypothesis and showed that the primary cilia of femoral tissue in diabetic SD rats were less than that in vehicle control group (Figure 4C,D). Protein levels analysis also found that Ac-α-tubulin and γ-tubulin were significantly decreased in femur (Figure 4E,F). These results verified that diabetes damages the primary cilia of pancreas as well as femur. Our previous research has confirmed that ICA could relieve osteoblast differentiation inhibition induced by high glucose (Figure 3), but the mechanism of action remains unclear. Here, we found that ICA could keep the morphology of primary cilia intact in a high glucose environment (Figure 4G), and protein level analysis showed that the expression of primary cilia structural proteins was hindered significantly by high glucose, while ICA could restore the expression effectively (Figure 4H,I). Based on these results, we speculate that ICA has therapeutic effects on bone loss caused by diabetes, likely by protecting osteoblast primary cilia to fulfil the function of information hub in bone development.

### 3.5. ICA Activates Hedgehog Signaling to Promote Osteoblast Differentiation by Maintaining Gli2’s Ciliary Localization

**Figure 5:**
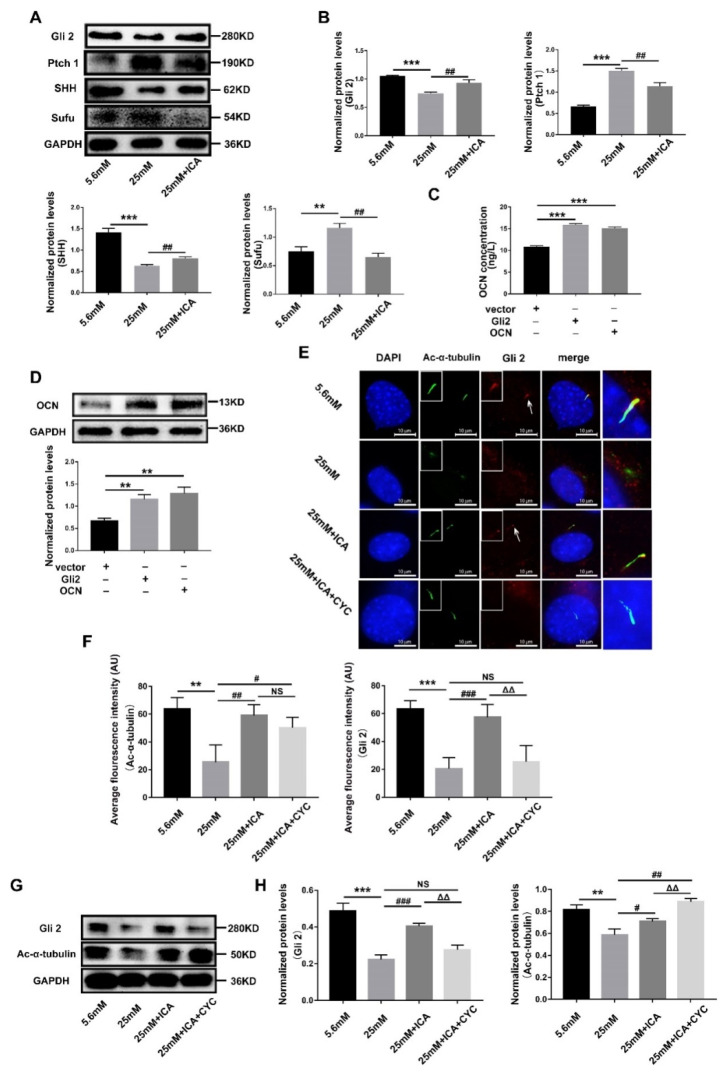
ICA activates Hedgehog signaling by maintaining primary cilia localization of Gli2. MC3T3-E1 cells were induced in different conditions for 3 days, extracted total cell protein. (**A**) Western blot detected Hedgehog signaling proteins Gli2, Ptch1, SHH and Sufu. (**B**) Quantitative analysis the protein expression level in (**A**). Overexpression Gli2 or OCN in HEK-293T cells, ELISA (**C**) and western blot (**D**) detected OCN protein. (**E**) Immunofluorescence observed primary cilia and Gli2 location. (**F**) Quantitative analysis average fluorescence intensity of Ac-α-tubulin and Gli2 in (**E**). Extracted total cell protein, (**G**) Western blot detected Gli2 and Ac-α-tubulin. (**H**) Quantitative analysis the protein expression level in (**G**). ** *p* < 0.01 and *** *p* < 0.001, compared with 5.6 mM group. ^#^ *p* < 0.05, ^##^ *p* < 0.01 and ^###^ *p* < 0.001, compared with 25 mM group. ^ΔΔ^ *p* < 0.01, compared with 25 mM + ICA group. NS: no significance.

Hedgehog signaling is essential for the development, homeostasis and regeneration of almost all organs in mammals, and downstream functions of PTCH1 and SMO depend entirely on primary cilia [45]. In the diabetic bone loss cell model, we detected Hedgehog signaling molecules and found that, compared with the 5.6 mM group, the protein expressions of Gli2, SHH in the 25 mM group were significantly reduced, while Ptch1 and Sufu were significantly increased (Figure 5A,B). These results suggest that high glucose inhibits Hedgehog signaling. Then, ICA addition reversed the inhibition by promoting expressions of Gli2 and SHH, and restraining Ptch1 and Sufu (Figure 5A,B), indicating that ICA could activate this pathway inhibited by high glucose. Given a prominent role of Gli2 in Hedgehog signaling, we transduced Gli2-expressing vector into HEK-293T cells to interrogate the functional importance for osteoblast. The results showed that Gli2 overexpression boosted OCN protein levels, and the efficiency was similar to that of the OCN overexpression group (Figure 5C,D). Subsequently, immunofluorescence detected the intracellular distribution of Gli2 under different conditions, and we found that Gli2 was normally co-localized with the ciliary axon protein Ac-a-tubulin (5.6 mM group). However, damage to the primary cilia caused by high glucose also leads to Gli2 disappeared (Figure 5E,F). These results suggest that primary cilia and Gli2 are “Fate Associates”. To further determine whether ICA benefits the primary cilia-dependent location of Gli2, we used Cyclopamine (CYC), a specific inhibitor of Hedgehog signaling, coupled with ICA to treat cells (25 mM + ICA + CYC group). The results showed that CYC suppressed Gli2 expression even though the primary cilia remained intact (Figure 5E–H), further demonstrating that primary cilia were indispensable for Gli2. This evidence suggests that Hedgehog signal is closely linked with primary cilia. Moreover, ICA could protect primary cilia from high glucose damage and assist Gli2 in anchoring in primary cilia.

### 3.6. ICA Alleviates the ROS Threat to Mitochondria and Primary Cilia

**Figure 6:**
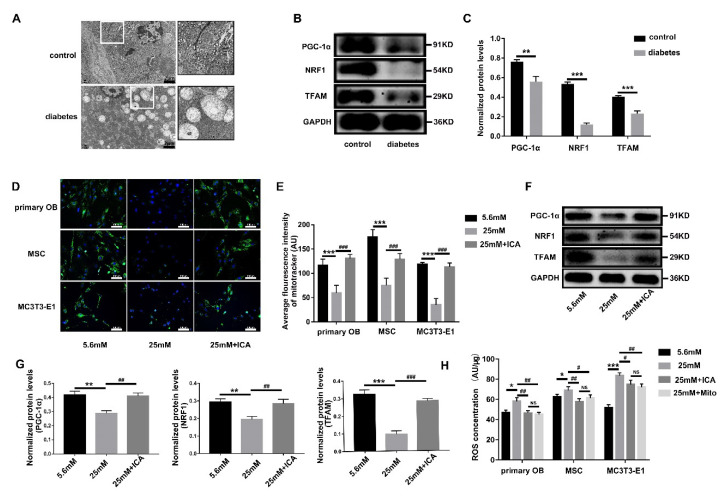
ICA inhibits ROS to maintain functional homeostasis of mitochondria and primary cilia. (**A**) Transmission electron microscope observed pancreatic tissues (scale bar: 2 μm, white box: mitochondria). (**B**) Western blot detected the expression of PGC-1α, NRF-1 and TFAM in the femoral protein. (**C**) Quantitative analysis the level of protein expression in (**B**). Three kinds of osteoblast cells were induced for 3 days, (**D**) immunofluorescence image of Mito-Tracker Green staining active mitochondria (green), DAPI staining the nucleus (blue), scale bar: 100 μm. (**E**) Quantitative analysis average fluorescence intensity of (**D**). (**F**) Extract total cell protein of MC3T3-E1, Western blot detected PGC-1α, NRF-1 and TFAM. (**G**) Quantitative analysis the protein expression level in (**F**). (**H**) The cell lysate of three kinds of osteoblasts was collected, DCFH-DA probe detected ROS content. * *p* < 0.05, ** *p* < 0.01 and *** *p* < 0.001, compared with 5.6 mM group. ^#^ *p* < 0.05, ^##^ *p* < 0.01 and ^###^ *p* < 0.001, compared with 25 mM group. NS: no significance.

Mounting clinical and experimental evidence shows that diabetes-related glucose fluctuations are particularly harmful to tissue regeneration and may cause metabolic disorders and mitochondrial dysfunction, characterized by increased oxidative stress [16]. Abundant swelling mitochondria were observed in pancreatic tissue of STZ-induced diabetic SD rats (Figure 6A). We interrogated mitochondria in the femur by detecting the marker proteins of mitochondrial oxidative metabolism function and found that protein levels of PGC-1α, NRF-1, and TFAM were inhibited significantly in diabetic SD rats (Figure 6B,C). The effects of high glucose on mitochondria were subsequently investigated in three osteoblast cell lines. Both Mito-Tracker Green immunofluorescence and protein levels analysis confirmed that high glucose impaired mitochondrial activity and function of osteoblasts. Intriguingly, adding ICA to high glucose medium remarkably restored the damaged mitochondria (Figure 6D–G). Mitochondria are the main organelles that generate ROS [46]. The superfluous ROS caused by chronic hyperglycemia in turn disrupted mitochondrial redox [47]. To further verify whether the promoting effect of ICA on mitochondria was related to counteracting ROS, we used DCFH-DA probe to detect ROS content in three induced osteoblasts lysates. The results showed that high glucose led to a significant increase in ROS, while ICA significantly reduce ROS production (Figure 6H). At the same time, we used Mito Tempo, a proven mitochondrion-specific scavenger of ROS, as a drug control, the results showed that ICA had a similar inhibitory effect on ROS as Mito Tempo (Figure 6H). More recently, Moruzzi et al. have shown that primary cilia damaged is the result of diabetes induced ROS excess and impaired mitochondrial metabolism [48]. These results suggest that ICA can rescue osteoblast differentiation under a high glucose environment because it inhibits ROS to maintain the functional homeostasis of mitochondria and primary cilia.

## 4. Discussion

The exact mechanism of diabetes induced bone complication is complex and can be caused by cellular abnormalities, matrix interactions, immune and vascular changes, and musculoskeletal maladaptation to chronic hyperglycemia [6]. Our findings confirmed that diabetes caused mitochondrial dysfunction characterized by increased ROS, which damaged the primary cilia of osteoblasts and inhibited differentiation-related signaling pathways.

To date, abundant evidence has supported that the initiation and development of diabetes are closely related to primary cilia [49,50]. One question we have is whether the morphological and functional impairment of primary cilia precede or are secondary to diabetes, but current research is inconclusive. Most of the current research on primary cilia-related diabetes focus on primary cilia regulating hormone secretion in pancreatic islet cells, and we speculate that the systemic metabolic disorders caused by progressive diabetes also damage the primary cilia in other tissues, directly causing diabetes-related bone, nerve, brain, retina, and other complications.

More than 600 proteins in reside in the primary cilia, which are critical for osteogenesis and chondrogenesis through a variety of signaling pathways, such as Hedgehog, Wnt/β-catenin, TGF-β, PDGF, and FGF [51,52]. Some diseases with cilia defect and/or malfunction caused by mutations of ciliary proteins are termed ciliopathies, with severe skeletal and craniofacial dysplasia [51]. Furthermore, it has been reported that some patients with ciliopathies, such as Bardet-Biedl and Alström Syndrome, commonly present with glucose intolerance and diabetes [53]. This evidence implies the linkage between ciliary dysfunction, bone defects, and diabetes. FOXO1 is an important positive regulator of bone development and regeneration [54]. Nevertheless, it plays an opposite role under diabetic pathological conditions [55]. Mechanistically, diabetes causes FOXO1 to shed from the promoter region of growth factors (such as TGF-β and VEGF) and to bind to the promoter region of pro-inflammatory and pro-apoptotic genes [56]. Interestingly, recent research suggests that deletion of FOX1 could rescue the cilia damaged by diabetes and promote fracture healing [51].

Mammalian Hedgehog signaling is entirely dependent on the primary cilia, and the two coevolved and adapted to each other to ensure that key signaling molecules are concentrated on the cilia for rapid response to low levels of ligands [57]. Gli protein can be proteolytically processed into three nuclear transcription factors, namely Gli1, Gli2, and Gli3, which respond to upstream Hedgehog ligand molecules [58]. In the absence of Hedgehog ligand, Gli protein remains a full-length unprocessed state and locates on the top of the primary cilia [59]. Advanced studies have confirmed that Hedgehog signaling can couple chondrogenesis with osteogenesis and regulate bone mass postnatally. Some bone diseases (such as skull abnormalities, polydactyly) are thought to be associated with abnormal Hedgehog signaling [60]. In this study, we confirmed that impairment of primary cilia induced by diabetes directly led to inhibition of Hedgehog signaling, characterized by reduced SHH/Gli2 expression and increased Ptch1/Sufu expression. We further confirmed the regulation of Hedgehog signaling on osteoblast differentiation and found that Gli2 significantly upregulated the expression of OCN, a marker of osteoblast differentiation.

The organism dysfunction induced by diabetes caused disability to respond or adapt to changes of metabolic demand, whereby glucose and fatty acids cannot be utilized flexibly [61], leading to the accumulation of toxic intermediates, especially excess ROS, damage DNA, lipids, endoplasmic reticulum, peroxisomes, mitochondria, and other cellular components and organelles [62]. Hyperglycemia-driven ROS increase is seen in both T1DM and T2DM, which is due to oxidative stress caused by an imbalance of oxidation and antioxidation, leading to the development of diabetic complications [63,64]. Our primary focus is on the role ROS plays in diabetic bone loss, and a large number of studies suggested that the main factors were that ROS promoted lipid peroxidation, reduced antioxidants, promoted bone resorption, and induced osteoblast apoptosis [65]. In addition, recent studies have confirmed that a lack of mitochondrial energy and ROS overproduction lead to cilia loss in diabetic nephropathy [48]. Ji et al. found that deletion of Prdx5, an antioxidant enzyme, could lead to abnormal ciliogenesis due to excess ROS [62], while decreased expression of several Prdx proteins was found in HFD-induced insulin resistance model mice [66]. These results are consistent with our findings that primary cilia are vulnerable targets of diabetes-induced ROS, but the mechanism of inducing ROS production needs to be further investigated.

Primary cilia can be used as an indicator of diabetes risk and treatment targets. Primary cilia integrate islet cell crosslinking and glucose homeostasis regulation. Specific deletion of islet β cell cilia not only impairs insulin secretion, but also affects glucagon and somatostatin secretion of proximal α and δ cells, leading to diabetes [67]. In the enrichment analysis of cilia-annotated genes in diabetic mice, Kluth et al. found that 81 human orthologs of the 327 differentially expressed genes were shared with diabetic patients, suggesting that decreased gene expression of islet cilia is a risk factor for predicting T2DM [33]. Acute kidney injury (AKI) induced by the antitumor drug cisplatin is associated with the shortening of the primary cilia of renal epithelial cells, resulting in the excretion of ciliary fragments and ciliary proteins into the urine as non-invasive biomarkers of AKI [68]. What’s more, several complications have been reported in association with damaged primary cilia, such as accelerating cystogenesis in diabetic kidney disease and defective diabetic fracture healing [53,69]. However, there is a lack of in vitro diagnostic methods to detect cilia-related markers in body fluids to predict disease progression, and specific tissue/organ origin classification of ciliary proteins is needed for future studies.

Glucose fluctuation induced ROS has been proposed as a therapeutic target for diabetic bone regeneration. As a native compound, ICA has antioxidant biological activity against ROS. Xia et al. demonstrated that ROS triggered by cisplatin chemotherapy led to cardiac injury through oxidative stress and activation of MAPKs and NF-κB signaling pathways, and ICA could alleviate these adverse effects [28]. The application of ICA in bone tissue engineering has great prospects. Liu et al. introduced icariin covalently into injectable hydrogels to promote chondrogenesis, which could be used as a candidate drug to prevent the progression of osteoarthritis [70]. The “gold standard” for treating non-union or slow healing fractures is bone grafting, whereas in dentistry and orthopedics the use of this treatment in patients with poorly controlled diabetes can lead to failure. In view of this, multiple logical tissue engineering strategies, in which engineered scaffolds are loaded with bioactive molecules and stem cells, have been extensively performed [71]. The PDLLA-PEG-PDLLA thermosensitive hydrogel designed by Wang et al. could continuously release metformin to remove excessive ROS, maintaining the natural bone healing cascade and promoting periodontal bone regeneration in diabetes [72]. A large number of studies have focused on the pathogenesis of ROS in diabetic complications. ICA is also widely used in the treatment of bone injury, but its research on diabetic bone complications is lacking. Our study reveals for the first time that ICA has a significant effect on removing excess ROS caused by diabetes and maintaining the functions of mitochondria and primary cilia, suggesting that ICA can be loaded onto bone tissue engineering scaffolds in the future for the treatment of bone injury in diabetic patients.

## 5. Conclusions

**Figure 7:**
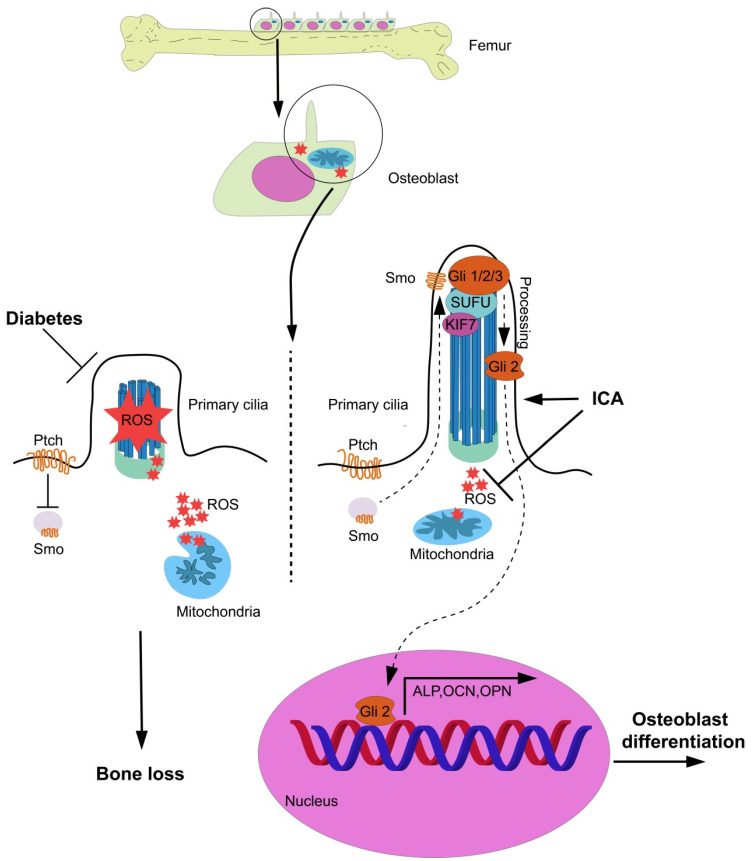
Scheme of the mode of ICA inhibits mitochondrial ROS production to rescue osteoblast differentiation by activate primary cilia/Gli2/osteocalcin signaling.

Our results confirmed that excessive ROS production induced by high glucose damaged osteoblast mitochondria and primary cilia. As a native antioxidant against ROS, ICA rescued the energy metabolism of mitochondria and biogenesis of primary cilia and facilitated osteoblast differentiation, suggesting that ICA can be used as a natural drug for the treatment of diabetes induced bone loss (Figure 7).

## Figures and Tables

**Table 1:** Multiple linear regression analysis for determining the risk factors of HbA1c.

Model	Unstandardized Coefficients		Standardized Coefficients	t	Significance	Collinearity Statistics	
	B	Std. Error	Beta			Tolerance	VIF
(Constant)	6.849	0.882		7.761	0.000		
LDL	0.635	0.100	0.253	6.369	0.000	0.971	1.030
ApoA	−1.421	0.396	−0.142	−3.584	0.000	0.968	1.033
P	1.315	0.445	0.116	2.953	0.003	0.985	1.015
ALP	0.025	0.003	0.284	7.249	0.000	0.992	1.008
UA	−0.005	0.001	−0.191	−4.791	0.000	0.959	1.043

Dependent Variable: HbA1c.

## Data Availability

Not applicable.
